# Interlayer exchange coupling in ferromagnetic semiconductor trilayers with out-of-plane magnetic anisotropy

**DOI:** 10.1038/s41598-019-41138-9

**Published:** 2019-03-18

**Authors:** Phunvira Chongthanaphisut, Seul-Ki Bac, Seonghoon Choi, Kyung Jae Lee, Jihoon Chang, Suho Choi, Sanghoon Lee, Moses Nnaji, X. Liu, M. Dobrowolska, J. K. Furdyna

**Affiliations:** 1 Department of Physics, Korea University, Seoul, 136-701 Korea; 2 Department of Physics, University of Notre Dame, Notre Dame, Indiana, 46556 USA

## Abstract

We report the observation of ferromagnetic (FM) and antiferromagnetic (AFM) interlayer exchange coupling (IEC) in GaMnAsP-based trilayer structures with out-of-plane magnetic anisotropy. Magnetization and anomalous Hall effect (AHE) measurements show well-resolved magnetization transitions corresponding to the two GaMnAsP layers. Minor loop measurements reveal a characteristic shift caused by IEC in all trilayer samples investigated. Interestingly, the FM IEC changes to AFM IEC for a trilayer with the thinnest (7 nm) top GaMnAsP layer as the temperature increases. The observation of temperature-induced transition of FM and AFM IEC in the same sample suggests the possibility of device applications by controlling the type of IEC in such GaMnAsP-based multilayers.

## Introduction

Interlayer exchange coupling (IEC) capable of stabilizing either parallel or antiparallel alignment between magnetizations in ferromagnetic multilayers, plays an important role in achieving giant magnetoresistance (GMR), an effect which has contributed immensely to spintronic technology^1–3^. Ferromagnetic GaMnAs, which is one of the most well-known diluted magnetic semiconductors (DMSs), has been widely investigated with an eye on using its unique ferromagnetic properties, such as carrier-dependent magnetism and magnetic anisotropy, for spintronic applications^4,5^. However, in comparison to the well-established metal-based ferromagnetic multilayer systems, the understanding of IEC in DMS-based multilayers still remains a challenge. For example, theoretical predictions of oscillation between antiferromagnetic (AFM) IEC and ferromagnetic (FM) IEC as a function of both non-magnetic spacer thickness and ferromagnetic layer thickness^6,7^ has been experimentally confirmed in diverse metal-based multilayer systems^8–10^. However, such oscillatory behavior has never been experimentally observed in DMS-based structures, even though a similar oscillatory behavior of IEC as in metal-based systems is predicted by theoretical studies^11,12^. We note additionally that, although the FM IEC itself is regularly observed in GaMnAs-based multilayers^13–16^, AFM IEC has only been seen in specially designed structures in which IEC is mediated by p-type non-magnetic GaAs spacers with specific thicknesses^17,18^.

Even though the IEC in magnetic multilayers is known to depend on various structural parameters (such as thickness of the non-magnetic spacer, barrier height, carrier concentration in the system, and on the magnetic layers themselves), investigations of this feature have so far mostly focused on the variation of properties of the spacer layer. Furthermore, the GaMnAs-based multilayer structures are normally grown on (001) GaAs substrates, which results in compressive biaxial strain of the GaMnAs layer, resulting in strong in-plane magnetic anisotropy in the GaMnAs film. However, magnetic films with an out-of-plane easy axis are especially desirable from the standpoint of applications because of their advantages owing to the possibility of achieving higher device densities, higher thermal stability for information storage, and lower critical current densities required for manipulating the magnetization^19^. In this context, IEC in multilayers composed of GaMnAs layers with out-of-plane easy axes has important implications for magnetic memory devices. IEC of GaMnAs-based systems with perpendicular magnetic anisotropy has recently been addressed using GaMnAs trilayers grown either on GaInAs or on ZnCdSe buffers^20–22^, both of which provide tensile strain in the GaMnAs layers, resulting in out-of-plane magnetic easy axes. These studies revealed the presence of FM IEC between the GaMnAs layers, with an exponential decay of the IEC strength as a function of spacer thickness^22^. However, until now there has been no observation of AFM IEC in GaMnAs-based structures with out-of-plane magnetic anisotropy.

Our present study addresses the IEC of GaMnAs-based structures with an out-of plane magnetic easy axis. For this purpose we have designed trilayer structures consisting of two GaMnAsP layers separated by a nonmagnetic GaAs:Be spacer. GaMnAsP has been selected for this purpose, since it is now well established that the incorporation of P in the GaMnAs crystal lattice results in tensile strain of the GaMnAsP layers grown on GaAs substrates^23–28^, thus inducing out-of-plane easy axes. The GaAs spacer was doped with Be to enhance the coupling strength as well as to increase the possibility of observing AFM IEC in the systems to be investigated^17,29–31^.We have also used different relative thicknesses of one GaMnAsP layer relative to the other in the three trilayers, which led to different coercivities in each of the trilayers. These differences in coercivity for the two GaMnAsP layers then allowed us to selectively reverse the magnetization in one of the two GaMnAsP layers of the trilayer relative to the other, thus providing an important handle for controlling and investigating the IEC between the GaMnAsP layers.

## Experiment

**Figure 1:**
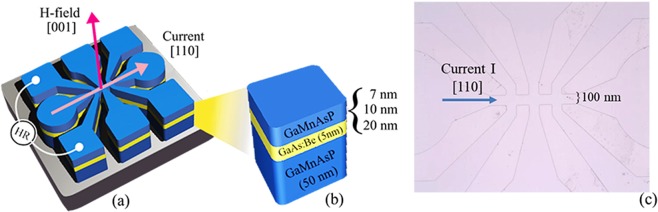
(**a**) Schematic diagram of the Hall device patterned on a GaMnAsP/GaAs:Be/GaMnAsP trilayer. Arrows show directions of the external field and current. (**b**) Structure of GaMnAsP/GaAs:Be/GaMnAsP trilayer grown on a GaAs substrate. (**c**) Optical image of the Hall device patterned on a GaMnAsP/GaAs:Be/GaMnAsP trilayer.

For the present study, a series of GaMnAsP/GaAs:Be/GaMnAsP trilayers were grown by molecular beam epitaxy (MBE) on (001) GaAs substrates. Prior to the growth of the GaMnAsP film, a 100 nm GaAs buffer layer was deposited on the substrate at 600 °C followed by deposition of a 10 nm low-temperature (LT)-GaAs buffer layer at 250 °C. On top of this GaAs buffer, the first Ga_1−*x*_Mn_*x*_As_1−*y*_P_*y*_ (bottom layer) with values of *x* and *y* near 0.04 and 0.1, respectively was grown to a thickness of 50 nm. A 5 nm GaAs spacer doped with Be was then deposited, with Be doping level estimated at ~10^20 ^cm^−3^ based on the temperature of the Be cell. Finally, the top Ga_1−*x*_Mn_*x*_As_1−*y*_P_*y*_ with the same values of *x* and *y* as in the bottom layer was deposited. The thicknesses of the top GaMnAsP layer in the series, *t*_top_, were 20, 10, and 7 nm, while the thicknesses of the GaAs spacer and the bottom GaMnAsP were kept the same in all three samples. Finally, a 5 nm GaAs:Be spacer thickness was used in all trilayers in order to increase the possibility of realizing AFM IEC, which was observed in GaMnAs/GaAs:Be/GaMnAs system with spacer thickness in the range of 3.5 to 7.1 nm. [29] For convenience, in discussing the properties of these samples we will refer to them simply as 20-nm, 10-nm, and 7-nm sample. A schematic figure of the trilayer structure displays in Fig. 1(b).

Two pieces were then cleaved from each multilayer for magnetization and transport measurements. The specimens were subsequently annealed using a hot plate at 200 °C for 30 minutes for transport measurements, and using a furnace at 260 °C for 60 minutes for magnetization measurement. Magnetization of the samples was measured as a function of magnetic field and temperature using a Quantum Design superconductor quantum interference device (SQUID) magnetometer. For transport measurements, Hall bars were patterned on each sample by photolithography and dry etching with widths of 100 µm and lengths of 1000 µm, with the long dimension aligned with [110] crystallographic direction of the GaAs substrate. Schematic diagram and an optical image of the Hall device are shown in Fig. 1(a,c), respectively. We have used 100 µA for Hall measurements in our experiment. Current density for the measurements can be calculated by using the cross-sectional area of the current channel at the position where the Hall voltage is measured. The current densities obtained for the 20, 10, and 7 nm samples are, respectively, 1.33 × 10^3^ A/cm^2^, 1.54 × 10^3^ A/cm^2^, and 1.61 × 10^3^ A/cm^2^. Magnetotransport measurements were carried out in a closed cycle cryostat using a cold finger whose temperature can be varied from 3 K to 300 K.

## Results and Discussion

**Figure 2:**
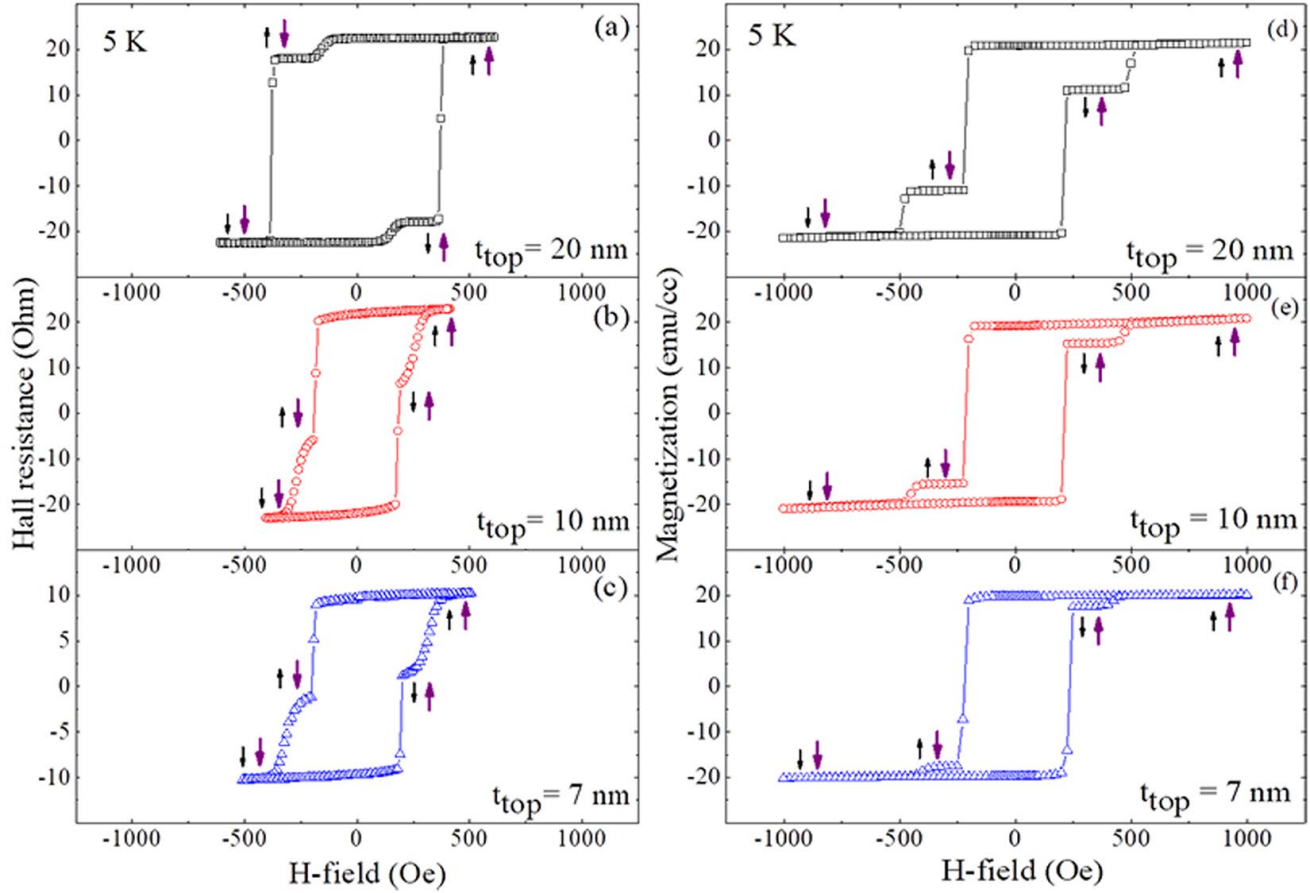
Hysteresis loops obtained during magnetization reversal. The 1^st^ and the 2^nd^ columns show Hall resistance and magnetization at 5 K, respectively. The data in the 1^st^, 2^nd^ and 3^rd^ rows are from the trilayer samples with top GaMnAsP thickness of 20, 10, and 7 nm, respectively. The small and large arrows at each plateau indicate directions of magnetization for the top and the bottom GaMnAsP layers, respectively.

To identify magnetization transitions and alignments in GaMnAsP layers, we performed anomalous Hall resistance (AHR) and SQUID measurements in a magnetic field applied perpendicular to the film plane (i.e., along the [001] crystallographic direction, as shown in Fig. 1(a)). The hysteresis loops obtained during the magnetization reversal are plotted in Fig. 2, where the 1^st^ and the 2^nd^ columns show Hall resistance measured at 3 K and magnetization measured at 5 K, respectively. The data in the 1^st^, the 2^nd^ and the 3^rd^ rows correspond to trilayers with top GaMnAsP thicknesses of 20, 10, and 7 nm, respectively. Hysteresis loops for all samples show a clear two-step transition, indicating different values of coercive field for the two GaMnAsP layers in each trilayer structure. The coercive field of each GaMnAsP layer can be unambiguously identified from the hysteresis obtained in SQUID measurements. The first transition (i.e., that occurring at the lower coercive field) shows much larger change in magnetization than the change seen in the second transition (i.e., that at the larger coercive field). Since the bottom GaMnAsP layer (50 nm) is always significantly thicker than the top GaMnAsP layer (20, 10, and 7 nm), the first transition in the hysteresis can thus be ascribed to the reversal of magnetization in the bottom GaMnAsP layer, and the second to the top layer.

Note that the AHR loop of the 20 nm sample shows a slow transition followed by the sharp transition, while both transitions are rather sharp in the hysteresis of magnetization measurement. Such difference may be caused by slightly different annealing conditions that were used for samples used in the two measurements, as described in the text. Thermal annealing enhances the magnetic uniformity as well as out-of-plane anisotropy of the GaMnAsP film. It is likely that this process was less effective in the case of samples used for the transport measurements owing to the lower annealing temperature and shorter annealing time (200 °C for 30 minutes, compared to 260 °C for 60 minutes in the case of samples used for magnetization measurements), especially for the sample with the thick (20 nm) top GaMnAsP layer. For this reason the bottom GaMnAsP layer in the 20 nm sample used in transport measurements may not have been fully annealed, thus resulting in slightly different transition behavior observed in AHR and in magnetization hysteresis loops.

**Figure 3:**
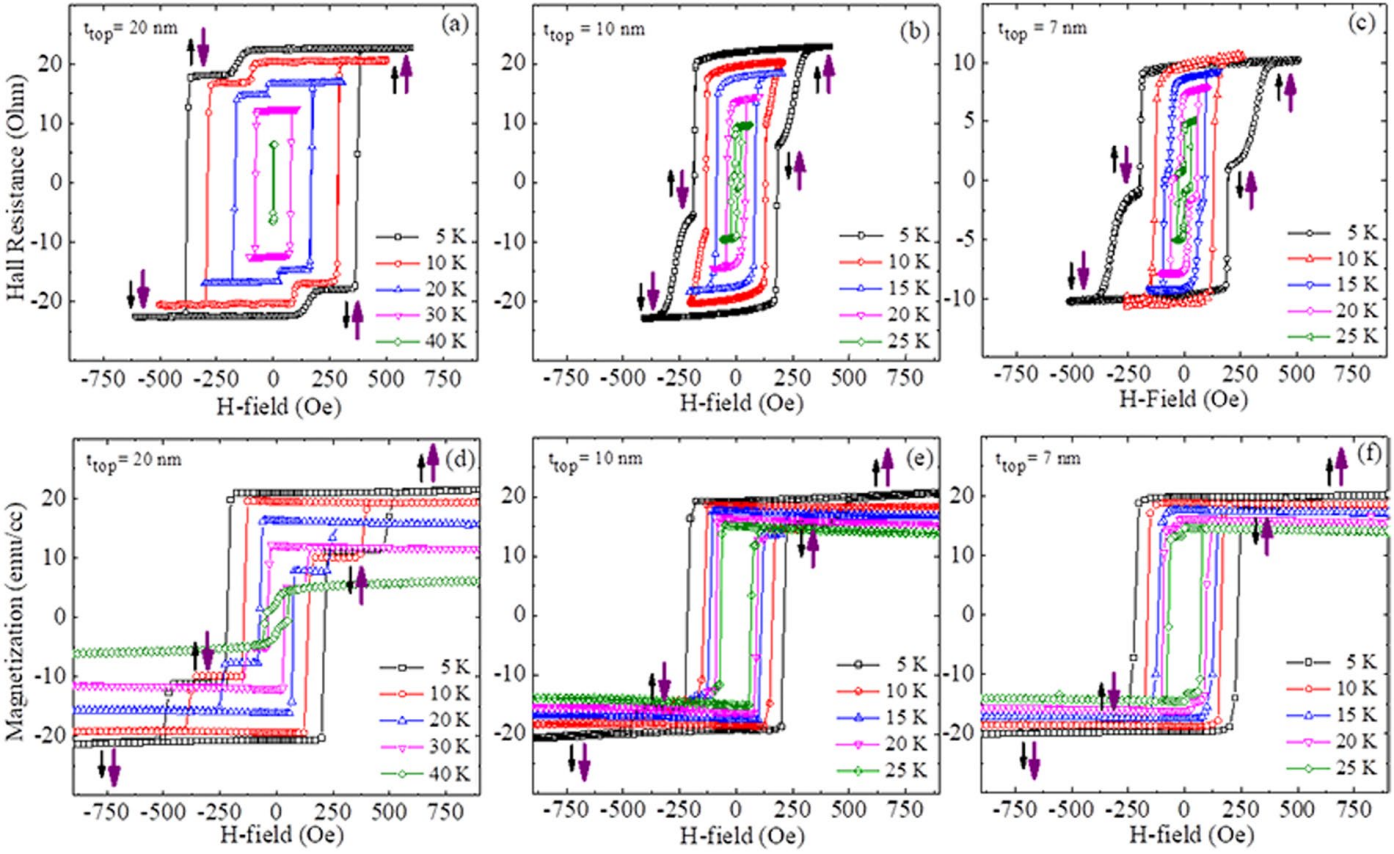
Hysteresis for the trilayer structures measured by Hall resistance (**a–c**) and magnetization (**d–f**) at various temperatures. The data obtained from trilayers with top GaMnAsP layer (t_top_) thicknesses of 20 nm, 10 nm, and 7 nm are plotted in the 1^st^, 2^nd^ and 3^rd^ columns, respectively. The small and large arrows at each plateau indicate directions of magnetization for the top and the bottom GaMnAsP layers, respectively.

The observed two-step transition in the magnetization reversal shows an interesting temperature behavior, seen in Fig. 3. For the sample with the 20 nm top GaMnAsP layer, the entire hysteresis systematically shrinks as the temperature increases and eventually disappears when the temperature increases to the Curie temperature of the GaMnAsP layer. Such temperature behavior is typically observed in trilayer structures consisting of two magnetic layers with different coercive fields^20^. Careful inspection of the hysteresis loops observed for the 7-nm sample, however, reveals an interesting feature that is different from the temperature behavior seen in the 20-nm sample. The two-step the hysteresis changes to a single-step loop as temperature increases, but the two-step behavior reappears as the temperature increases further (see Fig. 3(c,f)). This occurs because the coercive field of the top GaMnAsP layer (which displays a higher coercive field at low temperatures) decreases much faster with increasing temperature than that of the bottom GaMnAsP layer (which at low temperatures has a lower coercive field). At the temperature at which coercive fields of the two GaMnAsP layers become equal, the hysteresis becomes a single-step loop. As the temperature further increases, the coercive field of the top GaMnAsP layer becomes smaller than that of bottom GaMnAsP layer, thus resulting in the reappearance of the two-step hysteresis. Each transition can be traced as temperature varies. Representative data for this process are shown in supplementary materials. Note that the transitions order during the magnetization reversal is switched between top and bottom GaMnAsP layers in the high and the low temperature regions. This switching of the order of transitions with increasing temperature is also seen in the 10-nm sample, although it is less pronounced (see Fig. 3(b,e)).

**Figure 4:**
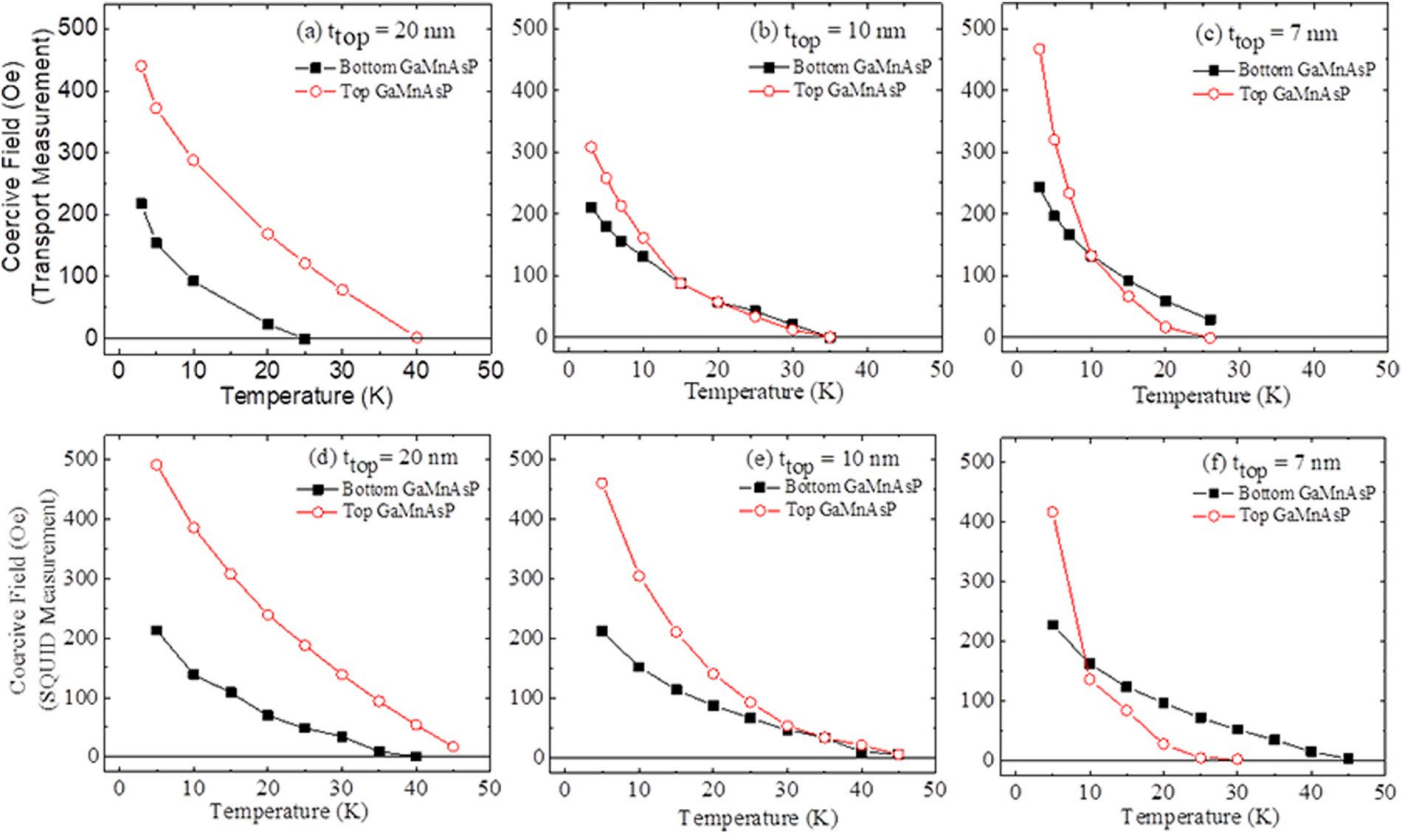
Temperature dependence of coercive fields of bottom and top GaMnAsP layers obtained from Hall measurement (**a–c**) and SQUID measurement (**d–f**). The 1^st^, 2^nd^ and 3^rd^ columns show data for samples with top layer thicknesses of 20 nm, 10 nm and 7 nm, respectively.

We have plotted the coercive fields for the two GaMnAsP layers for all samples as a function of temperature in Fig. 4, where black solid squares and red open circles represent coercive fields for the bottom and top GaMnAsP layers, respectively. All GaMnAsP layers show monotonically decreasing coercive fields as the temperature increases. Note, however, that their rates of decrease with increasing temperature are different for top and bottom GaMnAP layers, the coercive field of top layer decreases much faster than that of the bottom layer. Such difference in the rate of decrease of coercive field is especially significant in samples with thinner top layers, resulting in a crossing of coercive fields of the top and bottom GaMnAsP layers. Such temperature dependences of coercive field are consistently observed in both magneto-transport and SQUID measurements, as shown in the 1^st^ and 2^nd^ rows of Fig. 4.

**Figure 5:**
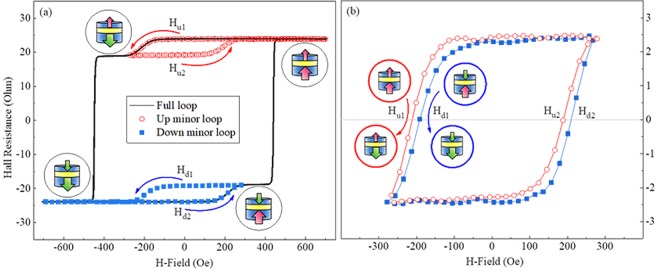
(**a**) Out-of-plane hysteresis loops showing the major (solid line) and minor (symbols) loop scans for the 20-nm sample. The first transition of the full loop scan arises when magnetization switches in one of GaMnAsP layers, causing an antiparallel magnetization configuration of the system. Curved arrows indicate the direction of minor loop scan. Insets show the schematic diagrams for the magnetization configurations at the corresponding positions. (**b**) Out-of-plane “Up” and “Down” minor loops are plotted together to find the value of the minor loop shift by calculating the average between (H_u1_ − H_d1_) and (H_d2_ − H_u2_).

In order to investigate interlayer exchange coupling (IEC) in our sample, we now focus on the Hall measurement results, which provide sufficiently fine steps in the field scans for identifying even very small shifts in transition fields that indicate changes in magnetization alignments in our trilayer samples. Specifically, we have performed minor loop scans to identify preferred magnetic alignments in our trilayer structures. Representative hysteresis loops obtained from the 20-nm sample at 3 K are shown in Fig. 5(a), where the major and minor loops are plotted with a black line and with symbols (red open circles and blue solid squares), respectively. The magnetic alignments between the two GaMnAsP layers are shown schematically with vertical arrows at each plateau. We obtained minor hysteresis loops at two different positions of the full hysteresis loop as shown in Fig. 5(a). One minor loop is recorded starting from parallel configuration of magnetization oriented along the positive-field direction (red open circles); and the other minor loop is measured starting from parallel configuration of magnetizations oriented along the negative field direction (blue solid squares). We refer to these two minor loops as “up minor loop” (Up-ML) and “Down minor loop” (Down-ML), respectively. In the Up-ML, the bottom GaMnAsP layer experiences the “Up-Down-Up” switching of magnetization direction, while the top GaMnAsP layer remains fixed along the “Up” direction. However, in the Down-ML, the bottom GaMnAsP experiences the “Down-Up-Down” direction switching, while the magnetization of the top GaMnAsP layer is fixed along the “Down” direction.

As will be shown, the shift of the two minor loops relative to each other provides a tool for quantitative determination of the strength of IEC between the two magnetic layers in the trilayer. To determine this shift, we plotted them together in Fig. 5(b) by adjusting only the center of Hall resistance values along the *y*-direction, while the center of the applied fields in *x*-direction remains unchanged. One can clearly see that the two minor loops do not overlap, but are shifted horizontally with respect to each other. The coercive fields of each minor loop during magnetization reversal are labeled as H_u1_, H_u2_, H_d1_ and H_d2_, as shown in Fig. 5. Note that the magnitude of H_u1_ is greater than that of H_d1_, while the magnitude of H_u2_ is smaller than H_d2_. This means that the transition of magnetization from parallel to antiparallel configuration is always harder than that from antiparallel to parallel. This indicates the presence of FM IEC between the GaMnAsP layers in the 20 nm system. In these Hall measurements, the step size in our field scans was 2.5 Oe for both minor and major loops. This allowed us to distinguish between Up-ML and Down-ML scans wider than a few Oe.

**Figure 6:**
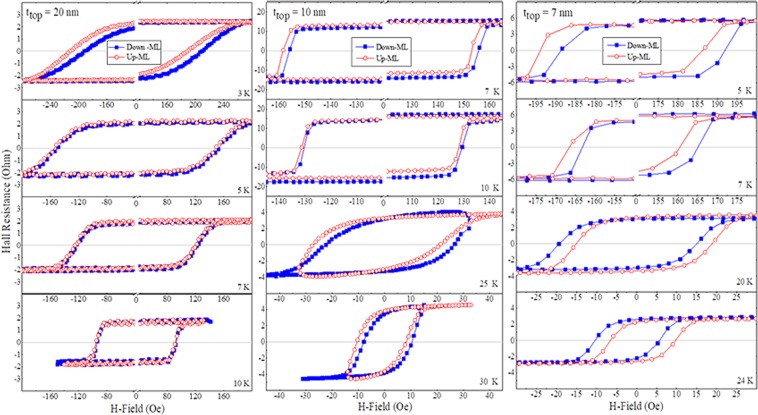
Temperature dependence of minor loops for the three samples. The data obtained from 20 nm, 10 nm, and 7 nm are plotted in the 1^st^, 2^nd^ and 3^rd^ columns, respectively. Red open circles and blue solid squares represent Up-ML and Down-ML, respectively.

We have measured the minor loops for all three samples at various temperatures. The results are plotted in Fig. 6, where the 1^st^, 2^nd^ and 3^rd^ columns represent, respectively, samples with 20, 10, and 7 nm top GaMnAsP layers. The red open and blue solid symbols in each panel represent Up-ML and Down-ML, respectively. All data measured at 3 K (see the 1^st^ row) show that Up-ML shifts to the left and Down-ML shifts to the right, indicating the presence of FM IEC between two GaMnAsP layers in all samples at this low temperature. The separation between the Up-ML and Down-ML in the 20 nm sample quickly drops with increasing temperature and becomes nearly zero near 10 K. Such monotonic temperature dependence of the IEC effect is normally observed in ferromagnetically coupled multilayers^22^. However, the 10- and 7-nm samples show a rather different temperature behavior, as seen in the 2^nd^ and 3^rd^ columns of Fig. 6. For example, there is a temperature region where one cannot discuss the IEC of the sample, since the coercive fields of the two GaMnAsP layers become nearly the same, and minor loops of the type shown in Fig. 5 cannot be measured. We will therefore discuss the IEC behavior for samples with 10 and 7 nm top GaMnAsP layers in two temperature regions, on either side of the temperature at which the coercive fields cross.

In the 10-nm sample (see 2^nd^ column of Fig. 6), separations between the Up-ML and Down-ML decrease with temperature in the low-temperature region (below 20 K), but reappear with the same direction and similar magnitude above the coercive field crossing temperature (i.e., above 20 K). This implies that FM IEC is comparable in both low and high temperature regions, quite different from the behavior of the 20 nm sample, which shows a monotonic decrease. Note that the shapes of the minor loops in the two temperature regions are rather different for 10 nm sample, i.e., transitions forming the minor loops at low temperatures are abrupt, while the transitions at high temperatures occur gradually over a wide field range. This is because at low temperatures the minor loops are formed by the sharp reversal of magnetization of the bottom GaMnAsP layer, while in the high temperature regime they are determined by the magnetization reversal in the top GaMnAsP layer.

The 7 nm sample, however, shows a different and more interesting IEC behavior in the temperature regions below and above coercive field crossing, as seen in the relative shifts of the Up-ML and Down-ML in the 3^rd^ column of Fig. 6. While the Up-ML shifts to the left and the Down-ML shifts to right at lower temperatures (see the top two panels in the 3^rd^ column of Fig. 6), the direction of their shift is clearly reversed above the crossing temperature, as seen in the bottom two panels. Since the direction of minor loop shifts reflects the preference of the two magnetic layers for parallel or antiparallel alignments, opposite minor loops shifts observed in the two temperature regions indicate that the IEC in the 7-nm sample changes from FM in low temperature region to AFM in higher temperatures. This temperature-induced change of IEC from FM to AFM has so far never been observed in any DMS-based multilayer system.

**Figure 7:**
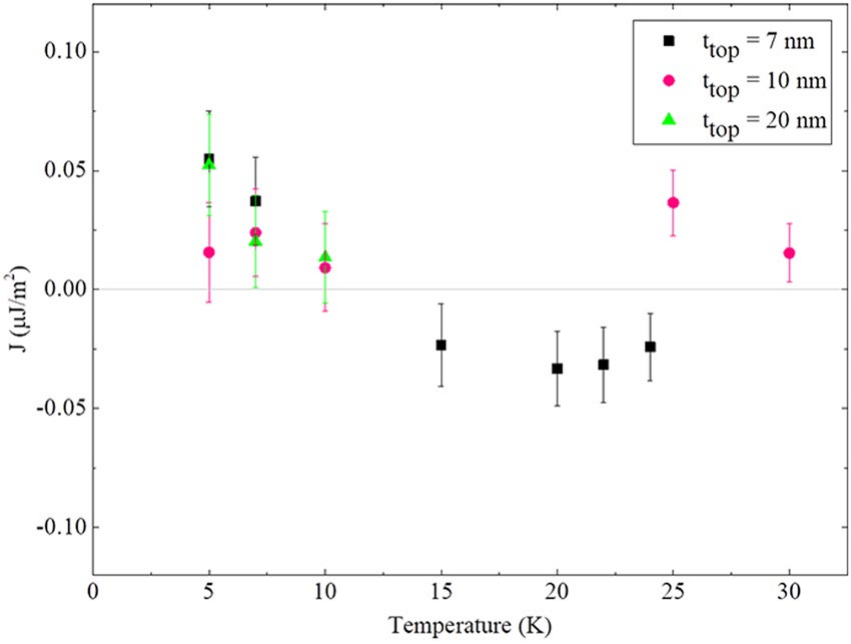
Temperature dependence of IEC strength for the three samples. The data obtained from 20 nm, 10 nm, and 7 nm samples are plotted with green triangles, pink circles, and black squares, respectively. Note that the sign change of J for the 7 nm sample with increasing temperature indicates conversion of IEC from FM to AFM in this sample.

Finally, we note that the shift of the minor loop from its center (obtained by measuring the coercive field difference between the Up-ML and Down-ML loops) provides a measure of the total coupling strength J_MLS_ given by the relation J = M_s_H_MLS_t_FM_, where M_s_ is spontaneous magnetization, H_MLS_ is the minor loop shift, and t_FM_ is the thickness of the ferromagnetic layer^14,32,33^. This provides a handle for quantifying the value of IEC ion our samples. In calculation, we have considered the uncertainties in switching field, magnetization, and thicknesses of the layers, and included their cumulative effect in the form of error bars in Fig. 7. The magnitude of J lies in the region between 0 and 0.06 *μ*J/m^2^, as seen in Fig. 7. The values of J obtained for our samples are somewhat smaller than those obtained from GaMnAs-based trilayer systems, which show J in the range between 0 and 9 *μ*J/m^2^ ^14,22^. The relatively weaker IEC obtained in the GaMnAsP-based systems may be due to the incorporation of P, which lowers the valence band relative to the GaAs spacer, so that the holes mediating the IEC feel a higher energy barrier between the two GaMnAsP layers. Note also that sign of J (positive or negative) indicates whether IEC is FM or AFM, respectively

In the 20 nm sample, J rapidly decreases with increasing temperature, which is in agreement with the conventional behavior of temperature-dependent IEC observed in earlier studies of GaMnAs-based systems^22^. Intriguingly, the FM IEC of the sample with 10 nm (pink solid circles) displays weak temperature dependence, and shows similar magnitudes of J in the two different temperature regimes.

For the 7 nm sample, the J is positive at low temperatures, indicating FM IEC, as expected. However, above the crossing of coercive fields of the two layers, J becomes negative, indicating that IEC became AFM in this sample. Since IEC in DMS multilayers, either FM or AFM, depends on the several parameters, such as carrier concentration and thickness of the spacer and thicknesses of the two magnetic layers, the cause of temperature-induced IEC change observed in our 7-nm sample cannot be identified at this time. However, note that the IEC conversion occurs as the temperatures increases over the coercive field crossing temperature. This implies that the observed reversal of IEC with increasing temperature may be related to the change in the order in which magnetization switches in the two GaMnAsP layers, which is a unique phenomenon observed in our GaMnAsP-based multilayers. Even though further systematic investigation is still necessary for understanding of the cause of the observed IEC conversion, the present study shows that both FM and AFM IEC can be realized in a single GaMnAsP-based multilayer and, importantly, that it can be switched by controlling appropriate tuning parameters, thus making it of interest for devices that involve manipulation of magnetization.

## Summary and Conclusions

We have used magnetization and Hall resistance to investigate IEC of trilayer structures comprised of two GaMnAsP ferromagnetic layers and a GaAs:Be spacer. Both magnetization and Hall measurements reveal that all GaMnAsP layer in our structures have out-of-plane magnetic anisotropy. Two-step hysteresis loops were observed for all trilayer samples during magnetization reversal in both SQUID and Hall resistance measurements, indicating that coercive fields of the two GaMnAsP layers in each trilayer are different. Significantly different temperature dependences of coercive fields were observed for GaMnAsP layers with different thicknesses. Importantly, in samples with top GaMnAsP layers of 7 and 10 nm, coercive fields of the two GaMnAsP layers show a crossing-over as the temperature increases. Minor hysteresis loop measurements, in which the magnetization of one GaMnAsP layer remains fixed while the other GaMnAsP layer experiences a reversal of its magnetization, show a shift indicating the presence of FM IEC in all samples when measured at low temperatures. However, in the sample with 7-nm top GaMnAsP layer, FM IEC changes to AFM IEC as the temperature increases above the coercive-field-crossing temperature. Even though the IEC conversion phenomenon from FM to AFM needs further investigation, the present study shows that both FM and AFM IEC can be realized in a single GaMnAsP-based multilayer, and that the IEC can be switched from FM to AFM by controlling appropriate tuning parameters, thus making the observed behavior of interest for spintronic device applications.

## Supplementary information

Interlayer exchange coupling in ferromagnetic semiconductor trilayers with out-of-plane magnetic anisotropy
