# Supplementary material for: Interlayer exchange coupling in ferromagnetic semiconductor trilayers with out-of-plane magnetic anisotropy
Source: Sci Rep. 2019 Mar 18;9:4740. doi: 10.1038/s41598-019-41138-9 (PMC6427040; doi:10.1038/s41598-019-41138-9)
Supplement: Supplementary file 1 — Interlayer exchange coupling in ferromagnetic semiconductor trilayers with out-of-plane magnetic anisotropy [file 41598_2019_41138_MOESM1_ESM.docx]

**Interlayer exchange coupling in ferromagnetic semiconductor trilayers with out-of-plane magnetic anisotropy**

**- Supplementary Material -**

Phunvira Chongthanaphisut^1^, Seul-Ki Bac^1,2^, Seonghoon Choi^1^, Kyung Jae Lee^1^, Jihoon Chang^1^, Suho Choi^1^, Sanghoon Lee^1*^, Moses Nnaji^2^, X. Liu^2^, M. Dobrowolska^2^, and J. K. Furdyna^2^

^1^*Department of Physics, Korea University, Seoul 136-701, Korea*

^2^*Department of Physics, University of Notre Dame, Notre Dame, Indiana 46556, USA*

* Correspondence: [slee3@korea.ac.kr](mailto:slee3@korea.ac.kr)

**Supplementary**

Since the observed two-step transition in the magnetization reversal of our trilayer system undergoes interesting changes as temperature increases, it is important to correctly identify the temperature dependence of switching fields of each GaMnAsP layer. In order to explain the detailed process of obtaining such temperature dependence, we use the data obtained from the 10 nm sample. All AHE hysteresis loops measured for this sample at different temperatures are plotted in the Fig. S1, where the scan for the positive field is magnified for clarity. One can clearly see two characteristic transitions for the two GMnAsP layers. The larger and smaller switching fields observed below 10 K correspond to the top and the bottom GaMnAsP layers, as identified from the magnetization measurements in the same temperature regions. We refer to these two transitions as “top” and “bot”, respectively, as marked in Fig. S1. Once the two transitions are identified, one can trace each transition systematically as marked at the switching field using the “top” and “bot” notation. Such traces clearly show that the switching fields of the two GaMnAsP layers cross each other at 10 K (i.e., the switching field for the top layer is larger than that of the bottom layer below 10 K, and the opposite is true for the temperature above 10 K.) Similar process was applied to the remaining samples to obtain the temperature dependence of their respective switching fields, as plotted in Fig. 4 in the main text.


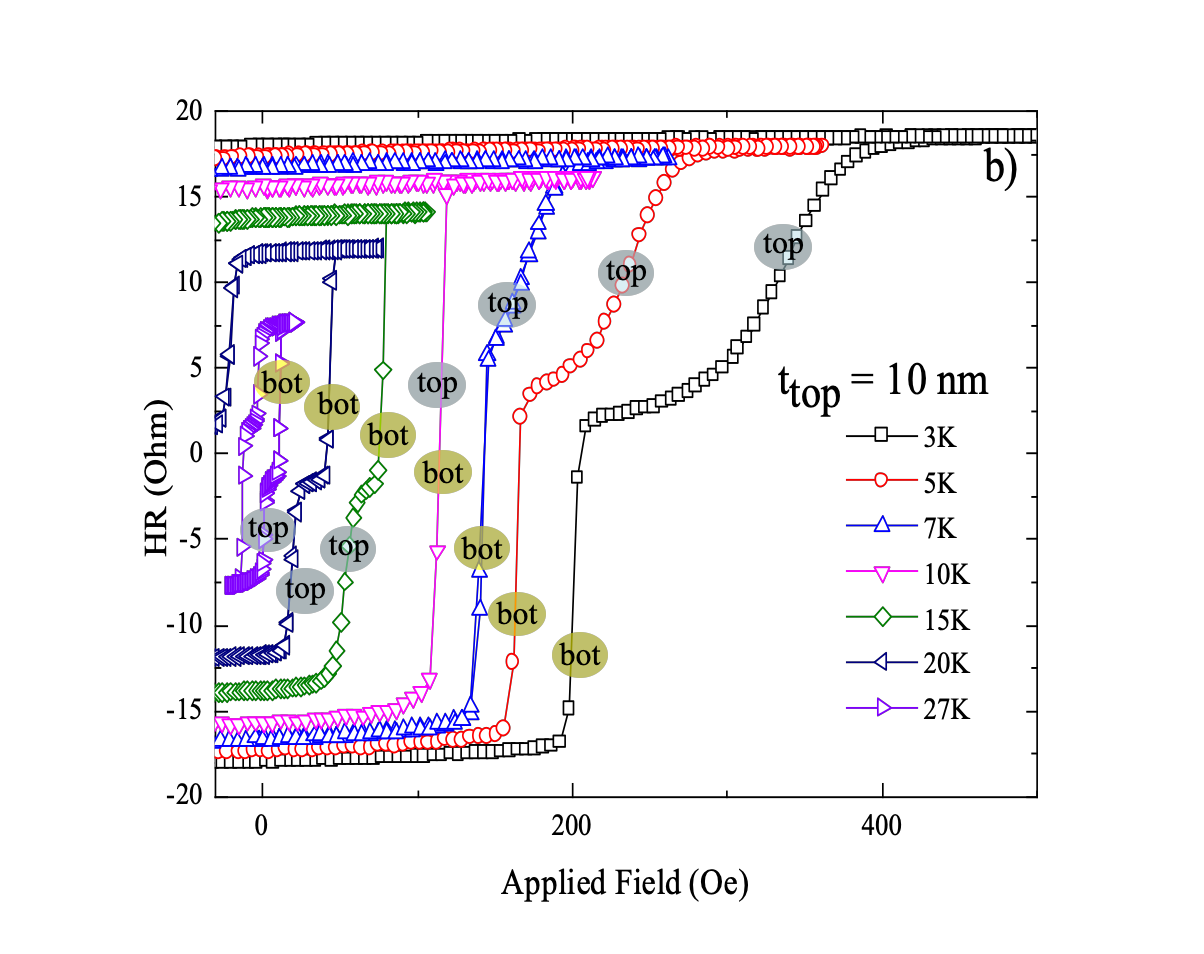


Fig. S1. Plot of AHE hysteresis measured at various temperatures for 10 nm sample. Transitions for the top and bottom GaMnAsP layers are marked as “top” and “bot”, respectively.
